# Psychrotolerant *Erwinia psychrophila* sp. nov. and *Erwinia magellanica* sp. nov. Isolated from Penguin Faeces

**DOI:** 10.1007/s00284-025-04670-8

**Published:** 2025-12-22

**Authors:** Ivo Sedláček, Pavla Holochová, Karel Sedlář, Eva Staňková, Mohammad Umair, Ondrej Šedo, Jitka Vives, Vendula Koublová, Dana Nováková, Pavel Švec

**Affiliations:** 1 Department of Experimental Biology, Faculty of Science, Czech Collection of Microorganisms, Masaryk University, Kamenice 5, Brno, 625 00 Czech Republic; 2 Faculty of Science, RECETOX, Masaryk University, Kamenice 5, Brno, 625 00 Czech Republic; 3 Department of Biomedical Engineering, Faculty of Electrical Engineering and Communication, Brno University of Technology, Technická 12, Brno, 616 00 Czech Republic; 4 Central European Institute of Technology, Masaryk University, Kamenice 5, Brno, 625 00 Czech Republic; 5 Global Change Research Institute of the Czech Academy of Sciences, Bělidla 986/4a, Brno, 60300 Czech Republic

**Keywords:** Magellanic penguin, *Erwinia*, Psychrotolerant, Taxonomy, Description

## Abstract

Our taxonomic study was aimed at characterizing enteric bacteria isolated from faecal samples of Magellanic penguins (*Spheniscus magellanicus*) collected in Cabo Virgenes, Patagonia, Argentina, in 2016 and 2017. Initial sequencing of the 16S rRNA gene assigned two psychrotolerant strains, P6884^T^ and P7711^T^, to the genus *Erwinia*. The closest phylogenetic relatives of the strains were *Erwinia billingiae* and *Erwinia endophytica* type strains, with 16S rRNA gene sequence similarity values between 98.4% and 98.9%. Whole-genome sequence analysis revealed that the strains analyzed could not be assigned to any validly named *Erwinia* species based on ANI and dDDH similarity values. Moreover, the ANI and dDDH similarities between the strains P6884^T^ and P7711^T^ genomes analyzed were 79.4% and 22.6%, respectively, indicating that they are distinct taxa. Further analysis using phenotyping and chemotaxonomy showed that the strains represent two newly discovered species of the genus *Erwinia*. The type strains P6884^T^ (= CCM 9357^T^ = LMG 33570^T^) representing *Erwinia psychrophila* sp. nov., and P7711^T^ (= CCM 9358^T^ = LMG 33569^T^) representing *Erwinia magellanica* sp. nov., were deposited in the Czech Collection of Microorganisms and BCCM/LMG Bacteria Collection. *Erwinia* species are typically associated with plants, fruits, and vegetables, and some species are important phytopathogens responsible for diseases in several economically important crops. The isolation of *Erwinia* species from penguin droppings shows that the microflora of penguin droppings, which can alter soil geochemistry in rookeries, may indicate yet unknown interactions within the Patagonian environment.

**Supplementary Information:**

The online version contains supplementary material available at 10.1007/s00284-025-04670-8.

## Introduction

Penguins are water birds that live mainly in the southern hemisphere. They spend most of their lives in the water and only come ashore during the breeding phase [1]. The Magellanic penguin (*Spheniscus magellanicus*) is a medium-sized penguin that breeds in the coastal areas of Patagonia. As important marine mesopredators, they feed in the water on small pelagic fish, squid, krill, and other crustaceans. Their diet may be responsible for the composition of their stomach microbiota, where the phyla *Bacillota* and *Pseudomonadota* predominate [2]. The examination of fresh faeces can provide information on the host microbiota in the gastrointestinal tract of penguins. It is a simple and non-invasive method of field sampling that enables research to be carried out within the relevant legal regulations [3, 4]. Penguin faeces can influence the ornithogenic soil microbiome directly by introducing microbes, and indirectly by altering soil geochemistry due to the rich organic matter in faeces [5].

The genus *Erwinia* belongs to the family *Erwiniaceae* [6], phylum *Pseudomonadota*, and comprises twenty species with a validly published name [7] (https://lpsn.dsmz.de/genus/erwinia, accessed May 23, 2025). *Erwinia* species are mainly plant pathogens or commensals, and many species that formerly belonged to the genus *Erwinia* are now assigned to the genera *Brenneria*, *Pantoea*, *Pectobacterium*, or *Winslowiella* [8]. *Erwinia* species cause spoilage of fresh vegetables and fruits due to their ability to produce pectin-lysing enzymes. They are often associated with a wide host range of plants, fruits, and vegetables, and some species are recognized phytopathogens responsible for diseases in several economically important crops. For example, *E. pyrifoliae* and *E. amylovora* are known to cause fire blight, which can affect the strawberry, raspberry plants, or apple and pear trees [9]. In the past, *E. tracheiphila* posed a threat to cucurbits crops in the United States [10]. Some *Erwinia* species are generally considered opportunistic pathogens for humans and animal hosts [11]. In humans, *Erwinia persicina* has been isolated from a urinary tract infection [12]. Other *Erwinia* species have been detected in several infections in humans [13], but infections of animals and humans by *Erwinia*-like microorganisms are rarely described [14]. Some strains of *Erwinia* are associated with insects, and these bacteria exploit insects as hosts and also as vectors due to the plant-to-plant infection cycle [15, 16].

The aim of this study was to investigate the faeces of Magellanic penguins, focusing on psychrotolerant Gram-stain negative fermenting rods, and to taxonomically characterize unidentified bacterial strains from the order *Enterobacterales*. In addition, we analyzed all genotypic and phenotypic features relevant to the classification of the novel species.

## Materials and Methods

### Sample Isolation and Reference Strains

Faecal samples of Magellanic penguins (*Spheniscus magellanicus*) were collected in January 2016 and February 2017 from the coast of Cabo Virgenes, Patagonia, Argentina (Fig. S1). Each fresh faecal sample was collected using a sterile Amies swab, stored in a refrigerator, and analyzed using culture-dependent analysis techniques. Endo agar, MacConkey agar, TCBS agar, Tergitol agar, and Yersinia selective agar (all Oxoid, U.K.) were used to isolate gut bacteria from faeces. The agar plates were inoculated directly with an Amies swab, and the plates were cultivated at 30 °C in aerobic conditions for four days. The isolates were sub-cultured on Tryptone Soya Agar to get a pure culture. Strain P6884^T^ was isolated on 5 January 2016, and strain P7711^T^ was isolated on 26 February 2017, both from MacConkey agar. The strains were maintained in a deep freezer at -70 °C until analyzed. The reference type strains *Erwinia billingiae* CCM 9268^T^, *Erwinia endophytica* CCM 9270^T^, *Erwinia persicina* CCM 3799^T^, *Erwinia amylovora* CCM 1114^T^, *Erwinia pyrifoliae* NBIMCC 8386^T^, *Erwinia oleae* DSM 23398^T^, *Erwinia aphidicola* DSM 19347^T^ and *Erwinia phyllosphaerae* JCM 34792^T^ were obtained from the Czech Collection of Microorganisms (CCM), Bulgarian National Collection for Microorganisms and Cell Cultures (NBIMCC), German Collection of Microorganisms and Cell Cultures (DSMZ), and Japan Collection of Microorganisms (JCM), and were used for the subsequent comparisons.

### Sequencing of the 16S rRNA Gene

Extraction of genomic DNA and amplification of the 16S rRNA gene were performed according to Sedláček et al. [17], and sequencing was performed at the Eurofins Genomics Core Facility, Germany. Nearly complete 16S rRNA gene sequences were compared in the EzBioCloud database [18]. Evolutionary analysis and tree construction were conducted in MEGA11 software [19]. The evolutionary history was inferred by using the Maximum Likelihood [20] and Neighbor-Joining methods [21], and the evolutionary distances were computed using the Kimura 2-parameter model [22]. Bootstrap test [23] based on 1000 replications was used. Initial tree(s) for the heuristic search were obtained automatically by applying Neighbor-Join and BioNJ algorithms to a matrix of pairwise distances estimated using the Maximum Composite Likelihood (MCL) approach, and then selecting the topology with the superior log likelihood value. The tree was drawn to scale, with branch lengths measured in the number of substitutions per site. All positions with less than 95% site coverage were eliminated, i.e., fewer than 5% alignment gaps, missing data, and ambiguous bases were allowed at any position (partial deletion option).

### Genome Sequencing and Analysis

DNA for whole-genome sequencing of strains P6884^T^ and P7711^T^ was isolated using a High Pure PCR Template Preparation Kit (Roche Diagnostics). Whole-genome sequencing of the analyzed strains was performed using a MiSeq instrument (Illumina, CA, USA). The library of fragments ranging from 300 to 500 bp was prepared using an Illumina DNA Prep; (M) Tagmentation Kit (Illumina, CA, USA), and Nextera™ DNA CD Indexes (Illumina CA, USA) were used as unique identifiers. The quality of sequencing reads was assessed using FASTQC v0.11.9, all lower-quality bases were removed, and adapters trimmed using trimmomatic v0.39 [24]. *De novo* assembly of the trimmed reads was performed with Unicycler assembler v0.4.8 [25]. The quality of the assembly was assessed using QUAST v5.0.2 [26], CheckM v1.2.2 [27], and CheckM2 v1.1.0 [28].

Initial similarity searches were performed online with the Type Strains Genome Server (TYGS) [29]. Average nucleotide identity values were calculated using OrthoANIu [30], and plotted as a heatmap with ComplexHeatmap [31], circlized [32], and viridis packages in RStudio [33–35]. Digital DNA-DNA hybridization (dDDH) values were calculated using the Genome to Genome Distance Calculator [29], using recommended Formula 2.

The genomes were annotated using the NCBI Prokaryotic Genome Annotation Pipeline (PGAP) [36]. In addition, the tool FGENESB [37] was used to recognize operons in both genomes. The pan-genome analysis of the genus *Erwinia* was performed using BPGA v1.3 [38], whereby the amino acid sequences were clustered using USEARCH [39] at an identity threshold of 50%. A total of 17 reference genomes of the genus *Erwinia* were obtained from the RefSeq database (accessed on 21st August 2025) [40] to define the core genome and perform phylogenomic analysis using the Neighbour-Joining algorithm implemented in BPGA. For a comprehensive analysis of the presented bacterial genomes, we performed functional annotation using eggNOG [41], together with Phage detection using PHASTEST [42], CRISPR (Clustered regulated interspaced short palindromic repeats) analysis using CRISPR-CasFinder [43], the creation of an antimicrobial resistance profile using the CARD (Comprehensive Antibiotic Resistance Database) Resistance Gene Identifier (RGI) [44] and the detection of plasmids using plasmidSPAdes [45], which was checked using the viralVerify tool.

### Genotyping Using RiboPrinter

Genomic relatedness between strains and reference cultures was determined by automated ribotyping with the restriction enzyme *Eco*RI in accordance with a previously published study [46].

### Phenotypic and Physiological Characterization

Basic phenotyping was performed using conventional tube and plate tests relevant for Gram-negative rods as previously described [47] with minor modifications – the optimal temperature, 30 °C, was used instead of 20 °C, and Tryptone Soya Medium instead of R2A medium was used as a base for growth tests with different conditions (temperature, pH, NaCl). Finally, the sensitivity of strains to antibiotic disks recommended for *Enterobacterales* was performed on Mueller-Hinton agar plates at 30 °C for 24 h in a frame with EUCAST guidelines v.14 (https://www.eucast.org). Additional biotyping with the ENTEROtest 24 (Erba Lachema, Czech Republic), API 50 CH with API 50 CHE Medium, API ZYM (bioMérieux, France), and Biolog Gen III MicroPlate (Biolog, USA) was performed according to the kit manufacturers’ recommendations, as described in a previous study [17].

### Matrix-assisted Laser Desorption Ionization-time of Flight Mass Spectrometry

Protein fingerprinting was performed by MALDI-TOF MS (Matrix-assisted laser-desorption/ionization time-of-flight mass spectrometry) using an Ultraflextreme instrument (Bruker Daltonics) after samples were treated with the ethanol/formic acid extraction protocol [48].

### Fatty Acid Methyl Ester Analysis (FAME)

For FAME, bacterial cultures were grown on TSA at 30 ± 2 °C for 24 h or 48 h to reach the late exponential growth stage according to the four-quadrant streak method [49]. The fatty acid methyl esters were extracted and analyzed using an Agilent 7890B gas chromatograph according to the standard protocol of the Sherlock MIDI Identification System (MIDI Sherlock version 6.2; MIDI database, RTSBA 6.21).

## Results

### 16S rRNA Sequence Analyses

**Fig. 1:**
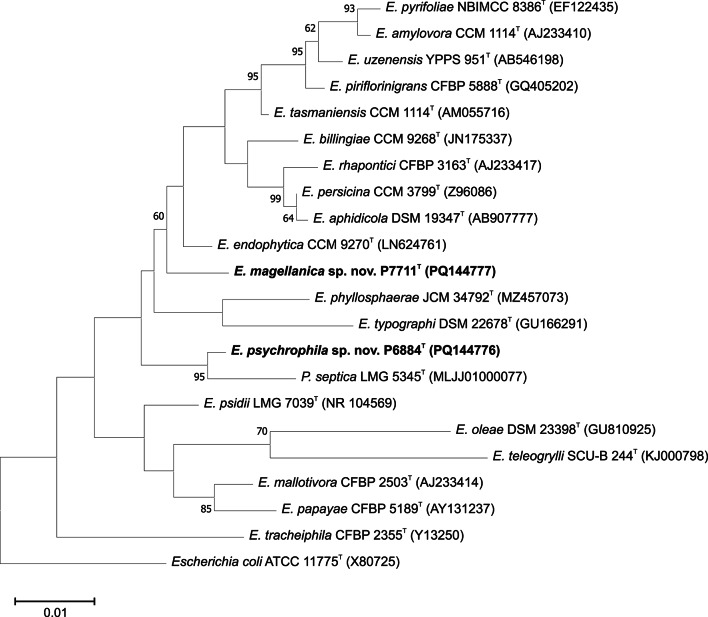
Tree based on 16S rRNA gene sequence comparison showing the phylogenetic position of strains P6884^T^ and P7711^T^ and the *Erwinia* and *Pantoea* reference type strains. Bootstrap probability values, percentages of 1000 tree replications greater than 50%, are indicated at branch points. There were a total of 1354 positions in the final dataset. *Escherichia coli* ATCC 11775^T^ was used as an outgroup. Bar, 0.01 substitutions per nucleotide position

Sequence analysis of the 16S rRNA gene in the EzBioCloud database assigned two psychrotolerant strains, P6884^T^ and P7711^T^, to the genus *Erwinia* and/or *Pantoea* (Fig. 1, Fig. S2). The closest phylogenetic relatives for strain P7711^T^ were *Erwinia billingiae* CCM 9268^T^ and *Erwinia endophytica*, CCM 9270^T^, or *Pantoea septica* LMG 5345^T^ and *Erwinia endophytica* CCM 9270^T^ for strain P6884^T^, respectively, with sequence similarities of the 16S rRNA gene between 98.4% and 98.9%. The GenBank accession numbers for 16S rRNA gene sequences of strains P6884^T^ and P7711^T^ are PQ144776 and PQ144777, respectively.

### Genome Features

To better resolve phylogenetic relationships, whole genome sequencing and *de novo* genome assembly of both analyzed strains were performed. This resulted in draft genomes of strains P6884^T^ and P7711^T^, available in the GenBank database under accession numbers JBCHVB000000000.1 and JBCHVC000000000.1, with total lengths of 4.2 Mbp and 4.5 Mbp, respectively, as summarized in Table 1. Although completeness percentages slightly below 95% calculated with CheckM seem rather low, some of the *Erwinia* species in the RefSeq database have even lower values, and CheckM2 predicted 100% completeness for both genomes. Predicted contamination is below for both genomes. The total lengths of the resulting genomes of P6884^T^ and P7711^T^ are close to the average length of 4.4 Mbp calculated using reference genomes of the genus *Erwinia* in the RefSeq database (see supplementary Table S1). Similarly, the number of predicted genes in both genomes, 3,941 and 4,213, was in the range of the total number of genes in fully complete genomes from 3,497 to 5,140. Similarly, calculated G+C DNA content 55.2% and 53.7% corresponded to other genomes in the RefSeq in the range from 50% to 55.5%.

**Table 1:** Genome features of strains P6884^T^ and P7711^T^

	P6884^T^	P7711^T^
**Genome Size**	4,244,786 bp	4,483,872 bp
**Coverage**	296×	388×
**Contigs**	34	23
**N50**	468,406	947,247
**L50**	3	2
**Completeness (CheckM/CheckM2)**	93.47%/100%	94.85%/100%
**Contamination (CheckM/CheckM2)**	3.24%/0%	2.91%/0.04%
**GC**	55.2%	53.7%
**Total No. of Genes**	3,941	4,213
**CDS**	3,860	4,134
**RNA**	81	79
**Complete rRNA**	1 (5S)	1 (5S)
**Partial rRNA**	2 (16S), 2 (23S)	1 (16S), 2 (23S)
**tRNA**	68	68
**ncRNA**	8	7

### Comparative Genome Analysis and Core Genome Phylogeny

**Fig. 2:**
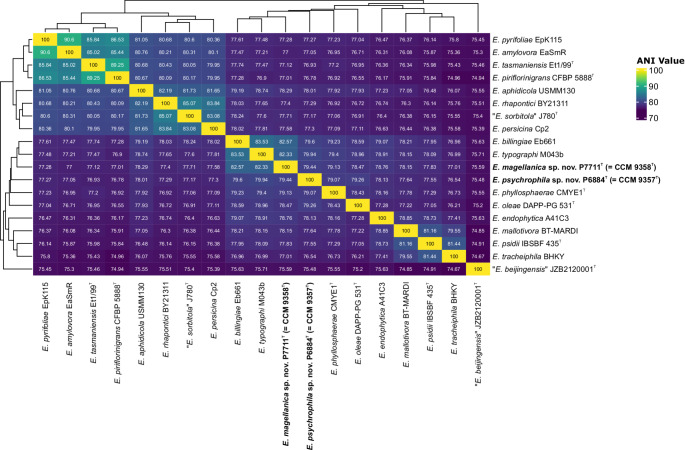
Heatmap showing ANI values between strains *Erwinia psychrophila* sp. nov. P6884^T^ and *Erwinia magellanica* sp. nov. P7711^T^, and the remaining *Erwinia* species

Initial whole-genome sequence analysis performed using TYGS showed no match to currently known type strains in the genus *Erwinia* and suggested that both strains belong to two novel species. Subsequent overall genome-relatedness index comparisons among the genomes of strains P6884^T^ and P7711^T^ and *Erwinia* species confirmed the taxonomic novelty of the analyzed strains at the species level. The ANI similarity between the two genomes of the strains analyzed was 79.4%, suggesting that they represent two distinct taxa, while it ranged from 75.5% to 82.6% between the two analyzed strains and reference genomes (Fig. 2). The dDDH similarity between strains P6884^T^ and P7711^T^ was 22.6%, while the similarities between both strains and related reference strains of *Erwinia* species with *Pantoea septica* ranged from 20.5 to 25.5% (Supplementary Table S2). All these data were below the threshold levels 95% (ANI) and 70% (dDDH) established for the species delineation [50], and confirmed that they represent two novel species of the genus *Erwinia*.

**Fig. 3:**
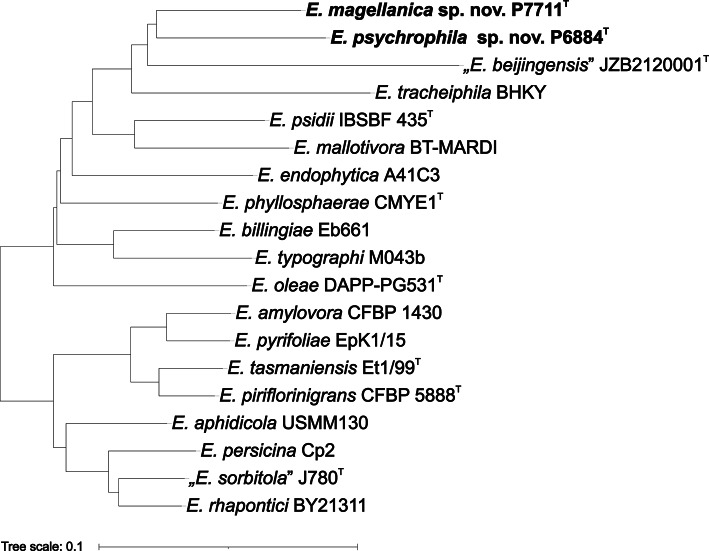
The phylogenetic position of analyzed strains within the genus *Erwinia* was obtained by phylogenomic analysis based on sequences of 1,472 core genes shared with 17 other representative genomes of *Erwinia* species from the RefSeq database. The core genome was identified with BPGA v 1.3.0 [38], with amino acid sequences clustered using USEARCH v11.0.667 [39], with an identity cut-off of 50%. The concatenated sequences of core genes were aligned with MUSCLE v3.8.31 [52], the distance matrix was calculated using the Kimura substitution model [22], and the final phylogenomic tree was reconstructed using the Neighbor-joining algorithm [21]

This conclusion was fully confirmed by the results of the pan-genome analysis (Fig. 3). Comparison of 17 reference genomes revealed that the representatives of the genus *Erwinia* share 1,473 genes that form the core genome of the genus. A phylogenomic tree reconstructed from the concatenated sequences of core genes separated the analysed species into two closely related branches of four species, while the remaining species show more distant phylogenetic relationships, similarly to ANI-based clustering. One of the two branches comprises pome fruit-associated species (*E. amylovora*,* E. pyrifoliae*,* E. piriflorinigrans*,* E. tasmaniensis*) [51] while the other group is associated with diverse ecological niches (*E. rhapontici*,* E. sorbitola*,* E. persicina*,* E. aphidocola*) [51].

### Functional Annotation and Analysis

Further analysis of the strain P6884^T^ genome identified 244 operons. Functional annotation of coding genes (CDS) identified a significant number of CDSs involved in numerous cellular processes categorized using COG (Cluster of Orthologous Groups) categories. Of the total 3,860 CDSs, 781 genes were classified as “S” (function unknown), accounting for about 21% of the total CDSs, 290 genes were classified as “K” (Transcription), and 224 genes were associated with “P” (transport and metabolism of inorganic ions) (Supplementary Table S3). In addition, 223 genes were found in the category “M” (Cell wall/membrane/envelope biogenesis), illustrating the diverse structural components of the bacterium. When analyzing the presence of phage regions in the strain P6884^T^ genome, three phage regions with different completeness were identified. Two regions with a size of 11.8 kb and 25.6 kb were labeled as incomplete, while one region with a size of 50.1 kb was labeled as intact. This could indicate a history of different phage interactions, with some prophages being potentially active while others are genomic remnants (Supplementary Table S4). We found phage-related genes such as integrase, terminase, portal, head, tail, and plate, indicating phage interactions and potentially active prophages. CRISPR analysis of the strain P6884^T^ genome revealed multiple CRISPR arrays with varying degrees of confidence. Of the four CRISPR arrays detected, only two were identified as high-confidence candidates. This demonstrates that strain P6884^T^ is able to adapt and defend itself against a variety of foreign threats using the CRISPR-Cas system (Supplementary Table S5). Antibiotic resistance profiling helped identify multiple antibiotic resistance genes using a rigorous paradigm that detects previously unknown variants of known antimicrobial resistance (AMR) genes and reveals the bacterium’s potential resistance to different antimicrobial agents. This helps to understand the clinical impact of the bacterium (Supplementary Table S6). The initial plasmid prediction indicated the existence of up to nine putative plasmids. However, further verification using the ViralVerify tool confirmed that a single sequence is the true plasmid, corresponding to contig six of the genome assembly.

Detection of operons in the strain P7711^T^ genome identified 305 operons, indicating coordinated gene expression and regulation in the bacteria. COG categorization of 4,134 CDSs revealed 808 genes associated with “S” (function unknown), 333 genes associated with “K” (Transcription), 299 genes associated with “P” (transport and metabolism of inorganic ions), and other genes distributed across different functional categories (Supplementary Table S3). Phage detection in the strain P7711^T^ genome identified three prophage regions within the genome that differed in completeness and size, with two regions of 38.2 kb and 40.7 kb classified as incomplete, and one region of 45.4 kb classified as intact (Supplementary Table S4). In phage recognition, genes such as integrase, terminase, portal, head, tail, and plate confirm possible phage-related activities that may play a role in the adaptability of bacterial strains and their genetic diversity. A CRISPR analysis was performed, and two CRISPR arrays were identified, but with very little evidence, indicating low confidence in the array. This could indicate that although some features of the CRISPR sequence are present, there is insufficient evidence to confirm a functional CRISPR-Cas system or highly diverse elements that are not currently recognized by any database (Supplementary Table S5). It could also indicate susceptibility to phage infection or dependence on alternative defense mechanisms. Antibiotic resistance profiling revealed the presence of different resistance genes according to a strict paradigm, emphasizing the antibiotic resistance potential of strain P7711^T^ (Supplementary Table S6). Finally, plasmid prediction indicated a single potential plasmid of approximately 5 kbp in length. After further verification with ViralVerify, the sequence corresponding to contig 13 of the genome assembly was confirmed to be a true plasmid.

### Ribotyping Analysis

Results obtained by ribotyping analysis with the RiboPrinter system showed that the ribotypes of the strains P6884^T^ and P7711^T^ were clearly separated from each other and from the ribotypes revealed by the remaining reference *Erwinia* type strains in the dendrogram (Fig. S3).

### MALDI TOF-MS Analysis

**Fig. 4:**
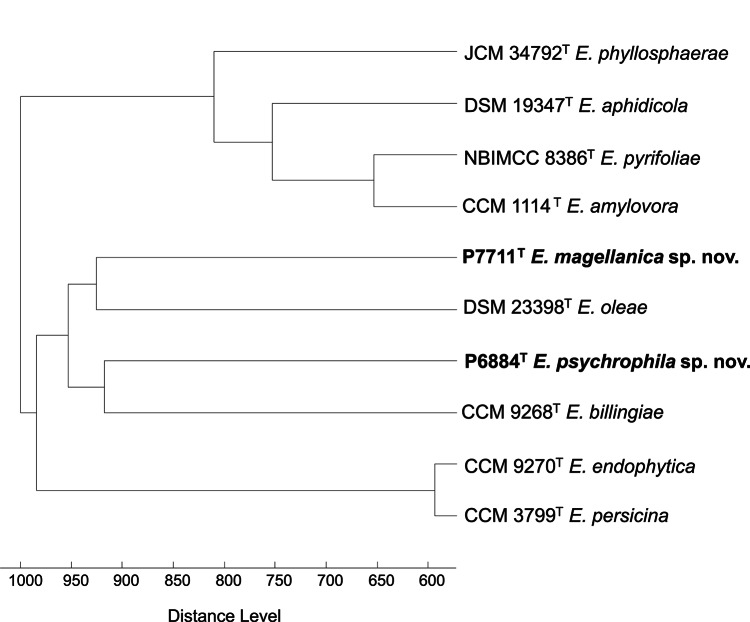
Dendrogram obtained by cluster analysis of MALDI-TOF mass spectra of *Erwinia psychrophila* sp. nov. P6884^T^, *Erwinia magellanica* sp. nov. P7711^T^, and closely related *Erwinia* species type strains generated with Biotyper 3.0 software (Bruker Daltonics) using Pearson’s product-moment coefficient as a similarity measure and the unweighted pair group average linked method (UPGMA) as a grouping method. The distance is expressed in relative units

In MALDI-TOF MS profiling, all tested reference strains had protein signals in the mass range of 2–12 kDa, while strains P6884^T^ and P7711^T^ had almost no consensus signals with the other *Erwinia* species. The protein fingerprints of isolates P6884^T^ and P7711^T^ did not show significant similarity to any of the Bruker Biotyper database entries (version 10.0, 9607 entries), with the highest scores equal to 1.151 (*Olsenella uli*) and 1.208 (*Arthrobacter woluwensis*), respectively (the score threshold for lowly confident identification is 1.700). This result is reflected in the cluster analysis, which placed both strains in very different branches of the dendrogram (Fig. 4).

### Whole-Cell Fatty Acids Analysis

Analysis of the fatty acid methyl esters showed that the fatty acid profiles of the two strains, P6884^T^ and P7711^T^, correspond to other phylogenetically related *Erwinia* species (Supplementary Table S7). The major fatty acids of the *Erwinia* genus are saturated fatty acids C_12:0_, C_14:0,_ and C_16:0_, as well as unsaturated fatty acids in summed features 3 (C_16:1_ ω7c/C_16:1_ ω6c) and summed features 8 (C_18:1_ ω7c/C_18:1_ ω6c) [51–54]. The presence of C_17:0_ cyclo was also reported, but its amount varied from species to species [55]. In all the cultures analyzed, we detected unknown fatty acids with an equivalent chain length (ECL) of 13.951 and ELC 14.502. Similar unknown fatty acids were also reported for *E. billingiae* [55]. The major fatty acids of strain P6884^T^ were summed feature 3 (35.6%), C_16:0_ (25.1%), and summed feature 8 (15.1%), followed by summed feature 2 (C_14:0_ 3OH/C_16:1_ iso I) (10.2%). Strain P7711^T^ had a similar fatty acid profile but with quantitative differences, especially in summed feature 3 and C_17:0_ cyclo. A comparison with the closest relatives revealed similar fatty acid profiles, confirming that both strains belong to the genus *Erwinia*. The quantitative differences in the major fatty acids distinguished them from their closest *Erwinia* species. relatives.

### Basic Phenotypic Characteristics

Strains P6884^T^ and P7711^T^ are motile, fermenting rods with positive catalase that produce light yellow endopigment. Both strains are halotolerant and grow well over a wide temperature range of 5–35 °C. Phenotypic identification with ENTEROtest 24 and the Biolog kit could not assign either strain to any known valid *Erwinia* species. Susceptible to aztreonam, cefixime, ciprofloxacin, doripenem, gentamicin, chloramphenicol, cotrimoxazole, nitroxoline, ofloxacin, and piperacillin. Susceptible, increased exposure to cefalexin and ceftazidime for strain P6884^T^, while susceptible for P7711^T^. Resistant to ampicillin and imipenem.

## Discussion

This study identified two novel bacterial strains, P6884^T^ and P7711^T^, isolated from the faeces of penguins as new members of the *Erwinia* genus. Unfortunately, genome assemblies of both analyzed strains contained no complete 16S RNA gene. This is caused by the limitation of short-read assembly with de Bruijn graphs that cannot resolve repetitive sequences. Since *Erwinia* species contain multiple copies of rRNA genes, very commonly even 7 copies of 16S rRNA, the copies in the analyzed strains caused their inability to be completely assembled. Nevertheless, the partial 16S rRNA genes of lengths 741 bp and 616 bp in the genome of the strain P6884^T^ and 1102 bp in the strain P7711^T^ matched the sequences previously obtained by Sanger sequencing with 100% sequence similarity.

Although strains P6884^T^ and P7711^T^ clustered together by the results of the pan-genome analysis (Fig. 3), they form clearly distinguishable taxa. Within the same clade of the phylogenomic tree, several mushroom and plant pathogens can be found, including “*Erwinia beijingensis*” (the name “*Erwinia beijingensis*” Xu et al. 2021 syn. “*Pantoea beijingensis*” Liu et al. 2013, was effectively but not validly published), isolated from the mushroom *Pleurotus eryngii* [56]– [57], *Erwinia tracheiphila*, cucurbit plants pathogen [58], *Erwinia psidii*, *Myrtaceae* plants pathogen [59], and *Erwinia mallotivora*, papaya dieback causal agent [60], confirming the evolutionary similarity of the two studied strains to phytopathogenic bacteria. However, other species within this clade were not yet associated with plant disease. *Erwinia endophytica*, originally isolated from healthy potato plants [61], and *Erwinia phyllospherae*, first isolated from pomelo leaves [53], were among the closest relatives of both studied strains within the 16S rRNA phylogenetic tree (Fig. 1). Several species were shown to have strong ecological ties to insects. *Erwinia typographi* is associated with the European spruce bark beetle *Ips typographus* [62], and *E. tracheiphila* is a cucurbit pathogen [63] depending on cucumber beetles for transmission and overwintering.

This broad ecological niche range corresponds to the relatively distant phylogenetic relationships observed within the clade. The variation in lifestyles among *Erwinia* spp. has been attributed to extensive horizontal gene transfer and plasmid exchange [64]. This genetic plasticity concerns mainly the accessory genes, excluding these effects from the core genome analysis and making this phylogenetic tree highly robust. The discrepancies between the BPGA phylogenomic tree, ANI-based clustering, and the 16S rRNA gene phylogenetic tree stem from different principles of the individual methods.

## Conclusion

The faeces of Magellanic penguins were colonized by previously unknown species for which the names *Erwinia psychrophila* sp. nov. and *Erwinia magellanica* sp. nov are proposed. The isolation of *Erwinia* species from the faeces of Magellanic penguins was unexpected, as *Erwinia* are normally recovered from a wide host range of plants, fruits, and vegetables, but can be considered opportunistic pathogens for humans and animal hosts, although rarely described. Therefore, this suggests that the microflora colonizing the gastrointestinal tract of penguins needs further investigation to fully understand the functions of *Erwinia* species and their impact on ornithogenic soil. It remains uncertain whether the members of the genus *Erwinia* isolated in this study are autochthonous microorganisms of the penguin gut or originate from their diet and/or environment.

The tests used to distinguish strains *E. psychrophila* sp. nov. P6884^T^ and *E. magellanica* sp. nov. P7711^T^ from the phylogenetically closest recognized *Erwinia* species are listed in Table 2.

**Table 2:** Differentiation of *Erwinia psychrophila* sp. nov. and *Erwinia magellanica* sp. nov. from related *Erwinia* species type strains

		Tube/plate tests^&^									API 50 CH^&^								
		37 °C	8% NaCl			ESL	YEP		VPT		MLT		SUC			GNT		LAC	
***E. psychrophila*** **sp. nov. P6884**^**T**^		+	w			+	w		+		+		-			+		w	
***E. magellanica*** **sp. nov. P7711**^**T**^		+	+			-	+		-		+		-			-		+	
*E. billingiae* CCM 9268^T^		-	-			+	-		+		+		-			+		-	
*E. endophytica* CCM 9270^T^		-	w			+	-		-		+		+			w		+	
*E. persicina* CCM 3799^T^		+	+			+	-		+		+		+			-		+	
*E. phyllosphaerae* JCM 34,792^T^		+	+			+	w		-		-		+			-		-	
*E. oleae* DSM 23,398^T^		+	+			+	w		-		-		-			+		-	
*E. aphidicola* DSM 19,347^T^		+	+			+	-		-		+		+			+		-	
*E. pyrifoliae* NBIMCC 8386^T^		-	-			-	-		-		-		+			-		-	
*E. amylovora* CCM 1114^T^		-	-			-	-		-		-		+			-		-	
Abbreviations: 37 °C, growth at 37 °C; 8% NaCl, growth in presence of 8% NaCl;																			
ESL, hydrolysis of esculin; YEP, yellow pigment on TSA; VPT, Voges-Proskauer																			
reaction (acetoin); MLT, acid from maltose; SUC, acid from sucrose; GNT, acid																			
from gluconate; LAC, acid from lactose.																			
^&^ read after 24–48 h																			
+, positive; -, negative; w, weak																			

## Species Protologue

### Description of *Erwinia psychrophila* sp. nov.

*Erwinia psychrophila* (*psy.chro’*phi.la. Gr. masc. adj. *psychros*, cold; Gr. masc. adj. *philos*, loving; N.L. fem. adj. *psychrophila*, cold-loving).

The cells are Gram-stain-negative, non-spore-forming rods that occur predominantly in pairs or irregular clusters and are motile. The colonies on tryptone soya agar (TSA) are circular, whole-edged, convex, smooth, glistening, and have a diameter of about 2–3 mm after 48 h of cultivation at 30 °C. Aerobic growth occurs on Endo agar, MacConkey agar, Tergitol-7 agar, R2A agar, Yersinia selective agar, Brain Heart Infusion agar, Columbia blood agar (without hemolysis), and Nutrient agar. No growth on the vibrio-selective TCBS agar at 30 °C. Growth occurs between 1 °C and 37 °C, but not at 42 °C. The cells grow well on TSA medium in the salinity range of 0% − 7% NaCl; the presence of 8% NaCl (w/v) inhibits growth. Good growth in the range of pH 5 to pH 6 only. Produces fermentative acid from glucose in the OF test medium, without gas production. Produces alkaline phosphatase, acid phosphatase, catalase, leucine arylamidase, pyrrolidonyl arylamidase, trypsin, and β-galactosidase. Does not produce arginine dihydrolase, cystine arylamidase, esterase (C4), esterase lipase (C8), lipase (C14), lysine and ornithine decarboxylase, naphthol-AS-BI-phosphohydrolase, N-acetyl-β-glucosaminidase, oxidase, phenylalanine deaminase, urease, valine arylamidase, α-galactosidase, α-chymotrypsin, α-fucosidase, α-glucosidase, α-mannosidase, β-glucosidase, and β-glucuronidase. Utilizes Simmons citrate and malonate as the carbon source. Hydrolyses ONPG. Does not produce indole and H_2_S. Does not hydrolyse casein, DNA, gelatine, lecithin, starch, Tween 80, and tyrosine.

Produces fermentative acid from glycerol, ribose, D-arabinose, L-arabinose, D-xylose, galactose, glucose, fructose, mannose, rhamnose, inositol, mannitol, N-acetyl glucosamine, amygdalin, arbutin, cellobiose, trehalose, xylitol, gentiobiose, D-fucose, D-arabitol, and 2-ketogluconate. Weakly produces acid from sorbitol, salicin, melibiose, and L-fucose. Does not produce acid from erythritol, L-xylose, adonitol, methyl D-xyloside, sorbose, dulcitol, methyl D-mannoside, methyl D-glucoside, inulin, melezitose, raffinose, amygdalin, glycogen, turanose, tagatose, L-arabitol, and 5-ketogluconate.

The positive utilization as a carbon source occurs for D-maltose, D-trehalose, D-cellobiose, gentiobiose, α-D-lactose, β-methyl-D-glucoside, D-salicin, N-acetyl-D-glucosamine, N-acetyl-β-D-mannosamine, α-D-glucose, D-mannose, D-fructose, D-galactose, D-fucose, L-fucose, L-rhamnose, inosine, D-mannitol, D-arabitol, myo-inositol, glycerol, D-glucose-6-PO4, D-fructose-6-PO4, glycyl-L-proline, L-alanine, L-aspartic acid, L-glutamic acid, L-histidine, L-serine, D-galacturonic acid, D-galactonic acid lactone, D-gluconic acid, D-glucuronic acid, glucuronamide, mucic acid, quinic acid, D-saccharic acid, methyl pyruvate, L-lactic acid, citric acid, L-malic acid, bromo-succinic acid, α-hydroxy-butyric acid, acetoacetic acid, propionic acid, acetic acid and formic acid. Dextrin, sucrose, D-turanose, stachyose, D-raffinose, D-melibiose, N-acetyl-D-galactosamine, N-acetyl neuraminic acid, 3-methyl glucose, D-sorbitol, D-aspartic acid, D-serine, gelatine, L-arginine, L-pyroglutamic acid, pectin, p-hydroxy phenylacetic acid, D-lactic acid methyl ester, α-keto glutaric acid, D-malic acid, Tween 40, γ-amino-butyric acid, β-hydroxy-D, L-butyric acid, and α-keto butyric acid does not serve as the carbon source for utilization.

Major cellular fatty acids are summed feature 3 (C_16 : 1_ ω7c/C_16: 1_ ω6c), C_16:0_ and summed feature 8 (C_18:1_ ω7c/C_18:1_ ω6c).

The type strain is P6884^T^ (= CCM 9357^T^ = LMG 33570^T^) isolated from the faeces of a Magellanic penguin (*Spheniscus magellanicus*). The GenBank accession number for the 16S rRNA of strain P6884^T^ is PQ144776. The draft genome of strain P6884^T^ has been deposited in GenBank/EMBL/DDBJ under accession number JBCHVB000000000, and the version described in this paper is JBCHVB000000000.1. The draft genome has a size of 4.2 Mbp with 34 contigs (N50 = 468,406 bp; L50 = 3). The genomic G + C content of the type strain is 55%.

### Description of *Erwinia magellanica* sp. nov.

*Erwinia magellanica* (ma.gel.la’ni.ca. N.L. fem. adj. *magellanica*, named after the Magellan Straits where penguins occur).

The cells are Gram-stain-negative, short rods that occur predominantly in pairs or irregular clusters and are motile. The colonies on Tryptone Soya agar (TSA) are yellow, circular, whole-edged, convex, smooth, glistening and have a diameter of about 2 mm after 48 h of cultivation at 30 °C. Aerobic growth occurs on Endo agar, MacConkey agar, Tergitol-7 agar, R2A agar, Yersinia selective agar, Brain Heart Infusion agar, Columbia blood agar (without hemolysis), and Nutrient agar. No growth on the vibrio-selective TCBS agar at 30 °C. Growth occurs between 5 °C and 35 °C, but not at 40 °C; weak growth at 1 °C after ten days. The cells grow well on TSA medium in the salinity range of 0%−9% NaCl; the presence of 10% NaCl (w/v) inhibits growth. Good growth in the range of pH 5 to pH 6 only. Produces fermentative acid from glucose in the OF test medium, without gas production.

Producess alkaline phosphatase, acid phosphatase, catalase, leucine arylamidase, pyrrolidonyl arylamidase, valine arylamidase (weak), trypsin (weak), and β-galactosidase. Does not produce arginine dihydrolase, esterase (C4), esterase lipase (C8), lipase (C14), cystine arylamidase, lysine and ornithine decarboxylase, naphthol-AS-BI-phosphohydrolase, N-acetyl-β-glucosaminidase, oxidase, phenylalanine deaminase, urease, α-chymotrypsin, α-galactosidase, α-glucosidase, α-mannosidase, α-fucosidase, β-glucosidase, and β-glucuronidase.

Utilizes Simmons citrate and malonate as the carbon source. Hydrolyses ONPG. Does not hydrolyse casein, DNA, gelatine, lecithin, starch, Tween 80, and tyrosine. Does not produce indole and H_2_S.

Produces fermentative acid from glycerol, L-arabinose, ribose, D-xylose, galactose, glucose, fructose, mannose, rhamnose, inositol, mannitol, N-acetyl glucosamine, arbutin, salicin, cellobiose, melibiose, trehalose, xylitol, gentiobiose, D-fucose, and D-arabitol. Weakly produces acid from sorbitol and inulin. Does not produce acid from D-arabinose, erythritol, L-xylose, adonitol, methyl D-xyloside, sorbose, dulcitol, methyl D-mannoside, methyl D-glucoside, amygdalin, melezitose, raffinose, amygdalin, glycogen, turanose, tagatose, L-fucose, L-arabitol, 2-ketogluconate, and 5-ketogluconate.

The positive utilization as a carbon source occurs for dextrin, D-maltose, D-trehalose, D-cellobiose, gentiobiose, α-D-lactose, β-methyl-D-glucoside, D-salicin, N-acetyl-D-glucosamine, N-acetyl-β-D-mannosamine, N-acetyl neuraminic acid, α-D-glucose, D-mannose, D-fructose, D-galactose, L-fucose, L-rhamnose, inosine, D-mannitol, D-arabitol, myo-inositol, glycerol, D-glucose-6-PO4, D-fructose-6-PO4, D-aspartic acid, D-serine, gelatine (weak), glycyl-L-proline, L-alanine, L-arginine, L-aspartic acid, L-glutamic acid, L-histidine, L-serine, D-galacturonic acid, D-galactonic acid lactone, D-gluconic acid, D-glucuronic acid, glucuronamide, mucic acid, quinic acid, D-saccharic acid, methyl pyruvate, L-lactic acid, citric acid, D-malic acid, L-malic acid, bromo-succinic acid, Tween 40, acetic acid, and formic acid. 3-methyl glucose, D-fucose, and acetoacetic acid are borderline. Sucrose, D-turanose, stachyose, D-raffinose, D-melibiose, N-acetyl-D-galactosamine, D-sorbitol, L-pyroglutamic acid, pectin, p-hydroxy phenylacetic acid, D-lactic acid methyl ester, α-keto glutaric acid, γ-amino-butyric acid, α-hydroxy-butyric acid, β-hydroxy-D, L-butyric acid, α-keto butyric acid, and propionic acid does not serve as a carbon source for utilization.

Major cellular fatty acids are summed feature 3 (C_16 : 1_ ω7c/C_16: 1_ ω6c), C_16:0_ and summed feature 8 (C_18:1_ ω7c/C_18:1_ ω6c).

The type strain is P7711^T^ (= CCM 9358^T^ = LMG 33569^T^) isolated from the faeces of a Magellanic penguin (*Spheniscus magellanicus*). The GenBank accession number for the 16S rRNA of strain P7711^T^ is PQ144777. The whole-genome sequence of strain P7711^T^ was deposited in DDBJ/ENA/GenBank under accession number JBCHVC000000000, and the version described in this paper is JBCHVC000000000.1. The resulting draft genome was 4.48 Mbp in size with 23 contigs (N50 = 947,247 bp; L50 = 2). The genomic G + C content of the type strain is 53.7%. 

## Electronic Supplementary Material

Below is the link to the electronic supplementary material.

Supplementary Material 1

## Data Availability

All sequencing data have been deposited in DDBJ/ENA/GenBank under the project accession No. PRJNA688026. The whole-genome sequences of *E. psychrophila* sp. nov. P6884^T^ and *E. magellanica* sp. nov. P7711^T^ have been deposited under accession numbers JBCHVB000000000 and JBCHVC000000000, respectively. The raw paired-end Illumina reads were deposited in the NCBI SRA database under accession numbers SRX25649884 and SRX25649885.

## References

[1] Meyer-Rochow VB, Gal J (2003) Pressures produced when Penguins pooh-calculations on avian defecation. Polar Biol 27:56–58

[2] Zeng Y-X, Li H-R, Han W, Luo W (2021) Comparison of gut microbiota between Gentoo and Adélie Penguins breeding sympatrically on Antarctic Ardley Island as revealed by fecal DNA sequencing, vol 13. Diversity, p 500

[3] Zeng Y-X, Li H-R, Han W, Luo W (2022) Molecular dietary analysis of Adélie (*Pygoscelis adeliae*) and Gentoo (*Pygoscelis papua*) Penguins breeding sympatrically on Antarctic Ardley Island using fecal DNA. Polar Biol 45:999–1011

[4] Dewar M, Wille M, Gamble A, Vanstreels RET, Bouliner T, Smith A et al (2023) The risk of highly pathogenic avian influenza in the Southern ocean: a practical guide for operators and scientists interacting with wildlife. Antarct Sci 35:407–414

[5] Guo Y, Wang N, Li G, Rosas G, Zang J, Ma Y et al (2018) Direct and indirect effects of Penguin faeces on microbiomes in Antarctic ornithogenic soils. Front Microbiol 9:552 29666609 10.3389/fmicb.2018.00552 PMC5891643

[6] Adeolu M, Alnajar S, Naushad S, Gupta S (2016) R Genome-based phylogeny and taxonomy of the *‘Enterobacteriales’*: proposal for *Enterobacterales* ord. nov. divided into the families *Enterobacteriaceae*, *Erwiniaceae* fam. nov., *Pectobacteriaceae* fam. nov., *Yersiniaceae* fam. nov., *Hafniaceae* fam. nov., *Morganellaceae* fam. nov., and *Budviciaceae* fam. nov. Int J Syst Evol Microbiol 66:5575–5599 10.1099/ijsem.0.001485 27620848

[7] Parte AC, Sardà Carbasse J, Meier-Kolthoff JP, Reimer LC, Göker M (2020) List of prokaryotic names with standing in nomenclature (LPSN) moves to the DSMZ. Int J Syst Evol Microbiol 70:5607–5612 32701423 10.1099/ijsem.0.004332 PMC7723251

[8] Brady C, Kaur S, Crampton B, Maddock D, Armold D, Denman S (2022) Transfer of *Erwinia toletana* and *Erwinia iniecta* to a novel genus *Winslowiella* gen. nov. as *Winslowiella toletana* comb. nov. and *Winslowiella iniecta* comb. nov. and description of *Winslowiella arboricola* sp. nov., isolated from bleeding cankers on broadleaf hosts. Front Microbiol 13:1063107 10.3389/fmicb.2022.1063107 PMC9712802 36466697

[9] Thomas BO, Lechner SL, Ross HC, Joris BR, Glick BR, Stegelmeier AA (2024) Friends and foes: bacteria of the hydroponic plant Microbiome. Plants 13:3069 39519984 10.3390/plants13213069 PMC11548230

[10] Rojas ES, Batzer JC, Beattie GA, Fleischer SJ, Shapiro LR, Williams MA et al (2015) Bacterial wilt of cucurbits: resurrecting a classic pathosystem. Plant Dis 99:564–574 30699691 10.1094/PDIS-10-14-1068-FE

[11] Kim J-S, Yoon S-J, Park Y-J, Kim S-Y, Ryu Ch, -M (2020) Crossing the Kingdom border: human diseases caused by plant pathogens. Environ Microbiol 22:2485–2495 32307848 10.1111/1462-2920.15028

[12] O´Hara C, Steigerwald AG, Hill BC, Miller JM, Brenner DJ (1998) First report of a human isolate of *Erwinia persicinus*. J Clin Microbiol 36:248–250 9431957 10.1128/jcm.36.1.248-250.1998 PMC124844

[13] Prod´homme M, Micol LA, Weitsch S, Gassend J-L, Martinet O, Bellini C (2017) Cutaneous infection and bacteremia caused by *Erwinia billingiae*: a case report. New Microbe New Infect 19:134–136 10.1016/j.nmni.2017.07.006 PMC5555272 28831302

[14] Muraschi TF, Friend M, Bolles D (1965) *Erwinia*-like microorganisms isolated from animal and human hosts. App Microbiol 13:128–131 10.1128/am.13.2.128-131.1965 PMC1058210 14325868

[15] Basset A, Khush RS, Braun A, Gardan L, Boccard F, Hoffmann JA et al (2000) The phytopathogenic bacteria *Erwinia carotovora* infects *Drosophila* and activates an immune response. PNAS 97:3376–3381 10725405 10.1073/pnas.070357597 PMC16247

[16] Skrodenyté-Arbačiauskiené V, Radžiuté S, Stunžénas V, Bůda V (2012) *Erwinia typographi* sp. nov., isolated from bark beetle (*Ips typographus*) gut. Int J Syst Evol Microbiol 62:942–948 21669921 10.1099/ijs.0.030304-0

[17] Sedláček I, Kwon S-W, Švec P, Mašlaňová I, Kýrová K, Holochová P et al (2016) *Aquitalea pelogenes* sp. nov., isolated from mineral peloid. Int J Syst Evol Microbiol 66:962–967 26637813 10.1099/ijsem.0.000819

[18] Chalita M, Kim YO, Park S, Oh HS, Cho JH, Moon J et al (2024) EzBioCloud: a genome-driven database and platform for Microbiome identification and discovery. Int J Syst Evol Microbiol 74:006421 38888585 10.1099/ijsem.0.006421 PMC11261700

[19] Tamura K, Stecher G, Kumar S (2021) MEGA 11: molecular evolutionary genetics analysis version 11. Mol Biol Evol 38:3022–3027 33892491 10.1093/molbev/msab120 PMC8233496

[20] Felsenstein J (1981) Evolutionary trees from DNA sequences: a maximum likelihood approach. J Mol Evol 17:368–376 7288891 10.1007/BF01734359

[21] Saitou N, Nei M (1987) The neighbor-joining method: A new method for reconstructing phylogenetic trees. Mol Biol Evol 4:406–425 3447015 10.1093/oxfordjournals.molbev.a040454

[22] Kimura M (1980) A simple method for estimating evolutionary rate of base substitutions through comparative studies of nucleotide sequences. J Mol Evol 16:111–120 7463489 10.1007/BF01731581

[23] Felsenstein J (1985) Confidence limits on phylogenies: an approach using the bootstrap. Evolution 39:783–791 28561359 10.1111/j.1558-5646.1985.tb00420.x

[24] Bolger AM, Usadel B (2014) Trimmomatic: a flexible trimmer for illumina sequence data. Bioinformatics 30:2114–2120 24695404 10.1093/bioinformatics/btu170 PMC4103590

[25] Wick RR, Judd LM, Gorrie CL, Holt KE (2017) Unicycler: resolving bacterial genome assemblies from short and long sequencing reads. PLoS Comput Biol 13:e1005595 28594827 10.1371/journal.pcbi.1005595 PMC5481147

[26] Gurevich A, Saveliev V, Vyahhi N, Tesler G (2013) QUAST: quality assessment tool for genome assemblies. Bioinformatics 29:1072–1075 23422339 10.1093/bioinformatics/btt086 PMC3624806

[27] Parks DH, Imelfort M, Skennerton CT, Hugenholtz P, Tyson GW (2015) CheckM: assessing the quality of microbial genomes recovered from isolates, single cells, and metagenomes. Genome Res 25:1043–1055 25977477 10.1101/gr.186072.114 PMC4484387

[28] Chklovski A, Parks DH, Woodcroft BJ, Tyson GW (2023) CheckM2: a rapid, scalable and accurate tool for assessing microbial genome quality using machine learning. Nat Met 20:1203–1212 10.1038/s41592-023-01940-w 37500759

[29] Meier-Kolthoff JP, Carbasse JS, Peinado-Olarte RL, Göker M (2022) TYGS and LPSN: a database tandem for fast and reliable genome-based classification and nomenclature of prokaryotes. Nucleic Acids Res 50:D801–D807 34634793 10.1093/nar/gkab902 PMC8728197

[30] Yoon S-H, Ha S, Lim J, Kwon S, Chun J (2017) A large-scale evaluation of algorithms to calculate average nucleotide identity. Antonie Van Leeuwenhoek 110:1281–1286. 10.1007/s10482-017-0844-4 28204908 10.1007/s10482-017-0844-4

[31] Gu Z, Eils R, Schlesner M (2016) Complex heatmaps reveal patterns and correlations in multidimensional genomic data. Bioinformatics 32:2847–2849. 10.1093/bioinformatics/btw313 27207943 10.1093/bioinformatics/btw313

[32] Gu Z, Gu L, Eils R, Schlesner M, Brors B (2014) Circlize implements and enhances circular visualization in R. Bioinformatics 30:2811–2812. 10.1093/bioinformatics/btu393 24930139 10.1093/bioinformatics/btu393

[33] Garnier S, Ross N, Rudis B, Filipovic-Pierucci A, Galili T et al (2023) sjmgarnier/viridis: CRAN release v0.6.3 (v0.6.3CRAN). 10.5281/zenodo.7890878. Zenodo

[34] RStudio: Integrated Development Environment for R. Posit Software, PBC, Posit team, Boston (2025) MA. URL http://www.posit.co/

[35] R Core Team (2023) R: A language and environment for statistical computing. R foundation for statistical computing. Vienna, Austria. https://www.R-project.org

[36] Tatusova T, DiCuccio M, Badretdin A, Chetvernin V, Nawrocki EP, Zaslavsky L et al (2016) NCBI prokaryotic genome annotation pipeline. Nucleic Acids Res 44:6614–6624 27342282 10.1093/nar/gkw569 PMC5001611

[37] Solovyev V, Salamov A (2011) Automatic annotation of microbial genomes and metagenomic sequences. In: Li RW (ed) Metagenomics and its applications in agriculture, biomedicine and environmental studies. Nova Science, pp 61–78

[38] Chaudhari NM, Gupta VK, Dutta C (2016) BPGA-an ultra-fast pan-genome analysis pipeline. Sci Rep 6:24373 27071527 10.1038/srep24373 PMC4829868

[39] Edgar RC (2010) Search and clustering orders of magnitude faster than BLAST. Bioinformatics 26:2460–2461 20709691 10.1093/bioinformatics/btq461

[40] O’Leary NA, Wright MW, Brister JR, Ciufo S, Haddad D, McVeigh R et al (2016) Reference sequence (RefSeq) database at NCBI: current status, taxonomic expansion, and functional annotation. Nucleic Acids Res 44:D733–745 26553804 10.1093/nar/gkv1189 PMC4702849

[41] Cantalapiedra CP, Hernández-Plaza A, Letunic I, Bork P, Huerta-Cepas J (2021) eggNOG-mapper v2: functional annotation, orthology assignments, and domain prediction at the metagenomic scale. Mol Biol Evol 38:5825–5829 34597405 10.1093/molbev/msab293 PMC8662613

[42] Wishart DS, Han S, Saha S, Oler E, Peters H, Grant JR et al (2023) PHASTEST: faster than PHASTER, better than PHAST. Nucleic Acids Res 51:W443–W450 37194694 10.1093/nar/gkad382 PMC10320120

[43] Couvin D, Bernheim A, Toffano-Nioche C, Touchon M, Michalik J, Néron B et al (2018) CRISPRCasFinder, an update of CRISRFinder, includes a portable version, enhanced performance and integrates search for Cas proteins. Nucleic Acids Res 46:W246–W251 29790974 10.1093/nar/gky425 PMC6030898

[44] Alcock BP, Huynh W, Chalil R, Smith KW, Raphenya AR, Wlodarski MA et al (2023) CARD 2023: expanded curation, support for machine learning, and resistome prediction at the comprehensive antibiotic resistance database. Nucleic Acids Res 51:D690–D699 36263822 10.1093/nar/gkac920 PMC9825576

[45] Antipov D, Hartwick N, Shen M, Raiko M, Lapidus A, Pevzner PA (2016) PlasmidSPAdes: assembling plasmids from whole genome sequencing data. Bioinformatics 32:3380–3387 27466620 10.1093/bioinformatics/btw493

[46] Švec P, Busse H-J, Sedlář K, Musilová J, Králová S, Staňková E et al (2023) Corynebacterium antarcticum sp. nov., Corynebacterium Marambiense sp. nov., Corynebacterium meridianum sp. nov., and Corynebacterium Pygosceleis sp. nov., isolated from Adélie Penguins (Pygoscelis adeliae). Syst Appl Microbiol 46:126390 36566621 10.1016/j.syapm.2022.126390

[47] Sedláček I, Holochová P, Sobotka R, Busse H-J, Švec P, Králová S et al (2021) Classification of violacein-producing psychrophilic group of isolates associated with freshwater in Antarctica and description of *Rugamonas violacea* sp. nov. Microbiol Spectr 9.e00452-21 10.1128/spectrum.00452-21 PMC8552646 34378950

[48] Friewald A, Sauer S (2009) Phylogenetic classification and identification of bacteria by mass spectrometry. Nat Prot 4:732–742 10.1038/nprot.2009.37 19390529

[49] Sasser M (1990) Identification of bacteria by gas chromatography of cellular fatty acids; MIDI technical note 101. Microbial ID, Inc., Newark, DE, USA

[50] Chun J, Oren A, Ventosa A, Christensen H, Aharal DR, da Costa MS et al (2018) Proposed minimal standards for the use of genome data for the taxonomy of prokaryotes. Int J Syst Evol Microbiol 68:461–466 29292687 10.1099/ijsem.0.002516

[51] Coutinho TA, Shin GY (2024) Erwinia. In: Trujillo ME, Dedysh S, DeVos P, Hedlund B, Kämpfer P, Rainey FA, Whitman WB (eds) Bergey’s Manual of Systematics of Archaea and Bacteria. John Wiley & Sons Inc. 10.1002/9781118960608.gbm01146.pub2

[52] Edgar RC (2004) MUSCLE: a multiple sequence alignment method with reduced time and space complexity. BMC Bioinformatics 5:113 15318951 10.1186/1471-2105-5-113 PMC517706

[53] Pan MK, Feng GD, Yao Q, Li J, Liu C, Zhu H (2022) *Erwinia phyllosphaerae* sp. nov., a novel bacterium isolated from phyllosphere of pomelo (*Citrus maxima*). Int J Syst Evol Microbiol 72:005316 10.1099/ijsem.0.005316 35416765

[54] Tao Y, Ge Y, Yang J, Song W, Jin D, Lin H et al (2023) A novel phytopathogen *Erwinia sorbitola* sp. nov., isolated from the faeces of Ruddy shelducks. Front Cell Infect Microbiol 13:1109634 36875519 10.3389/fcimb.2023.1109634 PMC9978198

[55] Mergaert J, Hauben L, Cnockaert MC, Swings J (1999) Reclassification of non-pigmented *Erwinia herbicola* strains from trees as *Erwinia billingia*e sp. nov. Int J Syst Bacteriol 49:377–383 10319458 10.1099/00207713-49-2-377

[56] Xu F, Yan H, Liu Y, Zhao S, Song S, Gu T et al (2021) A Re-evaluation of the taxonomy and classification of the type III secretion system in a pathogenic bacterium causing soft rot disease of *Pleurotus eryngii*. Curr Microbiol 78:179–189 33123750 10.1007/s00284-020-02253-3

[57] Liu Y, Wang S, Zhang D, Wei S, Zhao S, Chen S et al (2013) *Pantoea beijingensis* sp. nov., isolated from the fruiting body of *Pleurotus eryngii*. Antonie Van Leeuwenhoek 104:1039–1047 24013967 10.1007/s10482-013-0024-0

[58] Hauben L, Moore ER, Vauterin L, Steenackers M, Mergaert J, Verdonck L et al (1998) Phylogenetic position of phytopathogens within the *Enterobacteriaceae*. Syst Appl Microbiol 21:384–397 9779605 10.1016/S0723-2020(98)80048-9

[59] Caires N, Hermenegildo PS, Guimaräes LMS, Mafia RG, Zauza EÂV, Júnior NB et al (2019) Host range of erwinia psidii and genetic resistance of *Eucalyptus* and *Corymbia* species to this pathogen. For Pathol 49:e12527

[60] Md Saad M, Zainal-Abidin R-A, Hassan MA, Bakar NA (2022) New insights into host-pathogen interactions in Papaya dieback disease caused by *Erwinia mallotivora* in *Carica Papaya*. Eur J Plant Pathol 163:393–413

[61] Ramirez-Bahena MH, Salazar S, Cuesta MJ, Tejedor C, Igual JM, Fernandez-Pascual M et al (2016) *Erwinia endophytica* sp. nov., isolated from potato (*Solanum tuberosum* L.) stems. Int J Syst Evol Microbiol 66:975–981 26637820 10.1099/ijsem.0.000820

[62] Skrodenyté-Arbačiauskiené V, Radžiuté S, Stunžénas V, Büda V (2012) *Erwinia typographi* sp. nov., isolated from bark beetle (*Ips typographus*) gut. Int J Syst Evol Microbiol 62:942–948 21669921 10.1099/ijs.0.030304-0

[63] Shapiro LR, Paulson JN, Arnold BJ, Scully ED, Zhaxybayeva O, Pierce N et al (2018) An introduced crop plant is driving diversification of the virulent bacterial pathogen *Erwinia tracheiphila*. mBio: 9e01307-18 10.1128/mBio.01307-18 PMC6168856 30279283

[64] Liop P (2015) Genetic Islands in pome fruit pathogenic and non-pathogenic *Erwinia* species and related plasmids. Front Microbiol 6:874. 10.3389/fmicb.2015.00874 26379649 10.3389/fmicb.2015.00874 PMC4551865

