# Supplementary material for: Psychrotolerant Erwinia psychrophila sp. nov. and Erwinia magellanica sp. nov. Isolated from Penguin Faeces
Source: Curr Microbiol. 2025 Dec 22;83(2):93. doi: 10.1007/s00284-025-04670-8 (PMC12722478; doi:10.1007/s00284-025-04670-8)
Supplement: Supplementary file 1 — Supplementary Material 1 [file 284_2025_4670_MOESM1_ESM.pdf]

Psychrotolerant *Erwinia psychrophila* sp. nov. and *Erwinia magellanica* sp. nov. isolated from penguin faeces

Journal - Current Microbiology

Ivo Sedláček, Pavla Holochová, Karel Sedlář, Eva Staňková, Mohammad Umair, Ondrej Šedo, Jitka Vives, Vendula Koublová, Dana Nováková, Pavel Švec

Correspondence:

Prof. Ivo Sedláček, Ph.D.

ORCID 0000-0003-17170187X

e-mail: ivo@sci.muni.cz

Department of Experimental Biology, Czech Collection of Microorganisms, Faculty of Science, Masaryk University, Kamenice 5, 625 00 Brno, Czech Republic

Supplementary information

Table S1. Genomes used for the core-genome-based analyses.

Assembly Accession	Organism Name	Sequence Length (bp)	Assembly Level	GC (%)	No. of Genes	CheckM Completeness (%)	CheckM Contamination (%)
GCF_000026185.1	*Erwinia tasmaniensis* Et1/99^T	4067864	Complete Genome	53.5	3785	99.62	0.03
GCF_009738185.1	"*Erwinia sorbitola*" J780^T	4750253	Complete Genome	53	4343	97.56	5.18
GCF_022647505.1	"*Erwinia beijingensis*" JZB2120001^T	4107390	Complete Genome	50	3735	89.73	2.94
GCF_043228865.1	*Erwinia amylovora* EaSmR	3832351	Complete Genome	53.5	3497	98.04	0.22
GCF_019844095.1	*Erwinia persicina* Cp2	4802925	Complete Genome	55.5	4471	96.4	6.1
GCF_000196615.1	*Erwinia billingiae* Eb661	5372268	Complete Genome	55	4986	99.73	0.19
GCF_002952315.1	*Erwinia pyrifoliae* EpK1/15	4075681	Complete Genome	53.5	3811	95.79	0.59
GCF_020683125.1	*Erwinia rhapontici* BY21311	5164665	Complete Genome	54	4790	98.01	6.12
GCF_021365465.1	*Erwinia tracheiphila* BHKY	4958521	Complete Genome	50.5	5140	79.67	2.24
GCF_001050515.1	*Erwinia piriflorinigrans* CFBP 5888^T	3930655	Contig	53	3650	97.68	0.78
GCF_019132875.1	*Erwinia phyllosphaerae* CMYE1^T	4731600	Contig	54	4372	95.62	4.59
GCF_003846135.1	*Erwinia psidii* IBSBF 435^T	4503018	Scaffold	51.5	4190	89.27	4.14
GCF_000770305.1	*Erwinia oleae* DAPP-PG531^T	4744408	Contig	54.5	4705	92.24	2.96
GCF_037149315.1	*Erwinia aphidicola* USMM130	4837878	Contig	56.5	4567	98.45	4.03
GCF_000590885.1	*Erwinia mallotivora* BT-MARDI	4640069	Contig	52.5	4302	88.02	4.99
GCF_009295515.1	*Erwinia endophytica* A41C3	4227107	Scaffold	51.5	4032	85.99	3.48
GCF_000773975.1	*Erwinia typographi* M043b	5749517	Contig	55	5618	95.65	4.98

Table S2. The dDDH similarities (%) of ***Erwinia psychrophila* sp. nov. P6884^T^**, ***Erwinia magellanica* sp. nov. P7711^T^** and closely related *Erwinia* spp. and *Pantoea septica* reference genomes.

	**P6884^T**	**P7711^T**
	dDDH (%)	dDDH (%)
***E. psychrophila* P6884^T sp. nov.** (GCF 039023475)	100	22.6
***E. magellanica* P7711^T sp. nov.** (GCF 039023495)	22.6	100
E. amylovora CFBP 1430 (GCF 000091565)	21.4	21.2
E. aphidicola USMM 130 (GCF 037149315)	21.7	21.9
"*E. beijingensis*" JZB2120001^T (GCF 022647505)	21.0	21.0
E. billingiae Eb661 (GCF 000196615)	22.8	25.5
E. endophytica A41C3 (GCF 009295515)	21.8	21.9
E. oleae DAPP-PG531^T (GCF 000770305)	22.5	21.7
E. persicina Cp2 (GCF 019844095)	21.4	21.5
E. phyllosphaerae CMYE1^T (GCF 019132875)	22.7	22.6
E. pyrifoliae EpK1-15 (GCF 002952315)	21.4	21.5
E. tracheiphila BHKY (GCF 021365465)	20.9	21.0
Pantoea septica MGYG-HGUT-02423 (GCF 902386985)	20.6	20.5

Table S3. Summary of clusters of orthologous groups (COG) of ***Erwinia psychrophila* sp. nov. P6884^T** and ***Erwinia magellanica* sp. nov. P7711^T**.

COG category	Description	P6884^T		P7711^T	
		Gene count	Relative abundance	Gene count	Relative abundance
**V**	Defense mechanisms	41	1%	40	1%
**U**	Intracellular trafficking, secretion, and vesicular transport	66	2%	67	2%
**T**	Signal transduction mechanisms	91	2%	85	2%
**S**	Function unknown	781	21%	808	20%
**Q**	Secondary metabolites biosynthesis, transport, and catabolism	38	1%	39	1%
**P**	Inorganic ion transport and metabolism	224	6%	299	8%
**O**	Posttranslational modification, protein turnover, and chaperones	96	3%	83	2%
**N**	Cell motility	40	1%	74	2%
**M**	Cell wall/membrane/envelope biogenesis	223	6%	254	6%
**L**	Replication, recombination, and repair	159	4%	161	4%
**K**	Transcription	290	8%	333	8%
**J**	Translation, ribosomal structure, and biogenesis	194	5%	205	5%
**I**	Lipid transport and metabolism	72	2%	78	2%
**H**	Coenzyme transport and metabolism	177	5%	219	6%
**G**	Carbohydrate transport and metabolism	240	6%	201	5%
**F**	Nucleotide transport and metabolism	97	3%	128	3%
**E**	Amino acid transport and metabolism	237	6%	210	5%
**D**	Cell cycle control, cell division, chromosome partitioning	46	1%	48	1%
**C**	Energy production and conversion	207	6%	247	6%
**-**	Not in COG	128	3%	142	4%

Table S4. Phage regions in ***Erwinia psychrophila* sp. nov. P6884^T** and ***Erwinia magellanica* sp. nov. P7711^T**.

**P6884^T**							
**Contig**	**Region starts**	**Region end**	**Region length**	**Completeness (score)**	**Phage genes**	**GC %**	**Phage and Hypothetical protein %**
5	154576	166396	11.8Kb	incomplete (40)	integrase, head	50%	94%
6	181785	207476	25.6Kb	incomplete (40)	integrase, head	52%	96%
7	49073	99266	50.1Kb	intact (140)	injection, head, tail, coat, capsid, portal, terminase, lysis, integrase	52%	98%
**P7711^T**							
**Contig**	**Region starts**	**Region end**	**Region length**	**Completeness (score)**	**Phage genes**	**GC %**	**Phage and Hypothetical protein %**
1	28427	66689	38.2Kb	incomplete (60)	integrase, head	49%	97%
1	712592	753368	40.7Kb	incomplete (60)	integrase, transposase, tail	52%	97%
2	158532	203992	45.4Kb	intact (150)	integrase, terminase, portal, head, tail, plate	51%	96%

Table S5. CRISPR regions in ***Erwinia psychrophila* sp. nov. P6884^T** and ***Erwinia magellanica* sp. nov. P7711^T**.

**P6884^T**							
**Contig**	**CRISPR start**	**CRISPR end**	**CRISPR length**	**Conservation repeats (% identity)**	**Spacer**	**Conservation spacers (% identity)**	**Evidence level**
1	527492	527577	85	95.65217	1	100	1
2	343213	343899	686	60.71429	11	0	4
2	348356	350606	2250	78.57143	37	0	4
3	27556	27646	90	100	1	100	1
**P7711^T**							
**Contig**	**CRISPR start**	**CRISPR end**	**CRISPR length**	**Conservation repeats (% identity)**	**Spacer**	**Conservation spacers (% identity)**	**Evidence level**
3	68059	68128	69	96	1	100	1
3	86614	86827	213	90	3	20.51282	1

- Evidence **Level 1**: Arrays with less than four spacers, low conservation, least likely to be true CRISPRs.
- Evidence **Level 2**: Arrays with moderate repeat conservation, potential but uncertain CRISPR candidates.
- Evidence **Level 3**: Arrays with high repeat conservation and low spacer similarity, likely true CRISPRs.
- Evidence **Level 4**: Arrays with very well-conserved repeats and minimal spacer similarity, highly reliable true CRISPRs.

Table S6. Antibiotic resistance genes in ***Erwinia psychrophila*** **sp. nov. P6884**^{T} and ***Erwinia magellanica*** **sp. nov. P7711**^{T}.

**P6884**^{T}									
**Contig**	**Locus tag**	**Length**	**ARO**	**% identity of matching region**	**Detection criteria**	**Drug class**	**Resistance mechanism**	**AMR gene family**	**Antibiotic**
4	AAH446_11435	632	CRP	98.1	protein homolog model	macrolide antibiotic; fluoroquinolone antibiotic; penam	antibiotic efflux	resistance-nodulation-cell division (RND) antibiotic efflux pump	erythromycin; cloxacillin; oxacillin; norfloxacin
4	AAH446_11245	1184	*Escherichia coli* EF-Tu mutants conferring resistance to pulvomycin	96.18	protein variant model	elfamycin antibiotic	antibiotic target alteration	elfamycin resistant EF-Tu	pulvomycin
12	AAH446_19435	1184	*Escherichia coli* EF-Tu mutants conferring resistance to pulvomycin	95.67	protein variant model	elfamycin antibiotic	antibiotic target alteration	elfamycin resistant EF-Tu	pulvomycin
6	AAH446_15695	1538	KpnH	88.04	protein homolog model	macrolide antibiotic; fluoroquinolone antibiotic; aminoglycoside antibiotic; carbapenem; cephalosporin; penam; peptide antibiotic	antibiotic efflux	major facilitator superfamily (MFS) antibiotic efflux pump	erythromycin; gentamicin C; ciprofloxacin; spectinomycin; streptomycin; tobramycin; ceftazidime; ertapenem; piperacillin; azithromycin; imipenem; polymyxin B; polymyxin B1; polymyxin B2; polymyxin B3; polymyxin B4; norfloxacin; ticarcillin; gentamicin
6	AAH446_15685	530	emrR	85.71	protein homolog model	fluoroquinolone antibiotic	antibiotic efflux	major facilitator superfamily (MFS) antibiotic efflux pump	nalidixic acid

7	AAH446_15870	185	rsmA	85.25	protein homolog model	fluoroquinolone antibiotic; diaminopyrimidine antibiotic; phenicol antibiotic	antibiotic efflux	resistance-nodulation-cell division (RND) antibiotic efflux pump	trimethoprim; chloramphenicol
4	AAH446_12865	2408	*Morganella morganii* gyrB conferring resistance to fluoroquinolones	81.34	protein variant model	fluoroquinolone antibiotic	antibiotic target alteration	fluoroquinolone resistant gyrB	enoxacin; ciprofloxacin; levofloxacin; moxifloxacin; gatifloxacin; lomefloxacin; nalidixic acid; norfloxacin; ofloxacin; trovafloxacin; grepafloxacin; sparfloxacin; pefloxacin
1	AAH446_04010	431	fosA5	67.88	protein homolog model	fluoroquinolone antibiotic; aminoglycoside antibiotic; phosphonic acid antibiotic	antibiotic inactivation	fosfomycin thiol transferase	ciprofloxacin; gentamicin
1	AAH446_01860	3161	adeF	61.57	protein homolog model	fluoroquinolone antibiotic; tetracycline antibiotic	antibiotic efflux	resistance-nodulation-cell division (RND) antibiotic efflux pump	tetracycline
2	AAH446_07420	1775	*Haemophilus influenzae* PBP3 conferring resistance to beta-lactam antibiotics	52.03	protein variant model	cephalosporin; cephamycin; penam	antibiotic target alteration	Penicillin-binding protein mutations conferring resistance to beta-lactam antibiotics	ceftriaxone; ampicillin; cefaclor; cefotaxime; cefditoren; cefdinir
6	AAH446_14870	3116	adeF	41.7	protein homolog model	fluoroquinolone antibiotic; tetracycline antibiotic	antibiotic efflux	resistance-nodulation-cell division (RND) antibiotic efflux pump	tetracycline
1	AAH446_01400	332	qacJ	35.58	protein homolog model	disinfecting agents and antiseptics	antibiotic efflux	small multidrug resistance (SMR) antibiotic efflux pump	benzalkonium chloride

P7711[T]									
**Contig**	**Locus tag**	**Length**	**ARO**	**% identity of matching region**	**Detection criteria**	**Drug class**	**Resistance mechanism**	**AMR gene family**	**Antibiotic**
4	AAH450_14770	632	CRP	98.57	protein homolog model	macrolide antibiotic; fluoroquinolone antibiotic; penam	antibiotic efflux	resistance-nodulation-cell division (RND) antibiotic efflux pump	erythromycin; cloxacillin; oxacillin; norfloxacin
4	AAH450_14650	1184	*Escherichia coli* EF-Tu mutants conferring resistance to Pulvomycin	95.67	protein variant model	elfamycin antibiotic	antibiotic target alteration	elfamycin resistant EF-Tu	pulvomycin
1	AAH450_00045	1535	KpnH	87.43	protein homolog model	macrolide antibiotic; fluoroquinolone antibiotic; aminoglycoside antibiotic; carbapenem; cephalosporin; penam; peptide antibiotic	antibiotic efflux	major facilitator superfamily (MFS) antibiotic efflux pump	erythromycin; gentamicin C; ciprofloxacin; spectinomycin; streptomycin; tobramycin; ceftazidime; ertapenem; piperacillin; azithromycin; imipenem; polymyxin B; polymyxin B1; polymyxin B2; polymyxin B3; polymyxin B4; norfloxacin; ticarcillin; gentamicin
9	AAH450_20555	185	rsmA	85.25	protein homolog model	fluoroquinolone antibiotic; diaminopyrimidine antibiotic; phenicol antibiotic	antibiotic efflux	resistance-nodulation-cell division (RND) antibiotic efflux pump	trimethoprim; chloramphenicol
1	AAH450_00055	530	emrR	83.04	protein homolog model	fluoroquinolone antibiotic	antibiotic efflux	major facilitator superfamily (MFS) antibiotic efflux pump	nalidixic acid

4	AAH450_16720	2408	*Morganella morganii* gyrB conferring resistance to fluoroquinolones	80.85	protein variant model	fluoroquinolone antibiotic	antibiotic target alteration	fluoroquinolone resistant gyrB	enoxacin; ciprofloxacin; levofloxacin; moxifloxacin; gatifloxacin; lomefloxacin; nalidixic acid; norfloxacin; ofloxacin; trovafloxacin; grepafloxacin; sparfloxacin; pefloxacin
2	AAH450_08745	329	KpnF	77.06	protein homolog model	macrolide antibiotic; aminoglycoside antibiotic; cephalosporin; tetracycline antibiotic; peptide antibiotic; rifamycin antibiotic; disinfecting agents and antiseptics	antibiotic efflux	small multidrug resistance (SMR) antibiotic efflux pump	erythromycin; streptomycin; tetracycline; cefepime; ceftriaxone; rifampin; colistin A; colistin B; triclosan; benzalkonium chloride; chlorhexidine
1	AAH450_05360	3161	adeF	61.66	protein homolog model	fluoroquinolone antibiotic; tetracycline antibiotic	antibiotic efflux	resistance-nodulation-cell division (RND) antibiotic efflux pump	tetracycline
4	AAH450_16055	398	FosA8	56.62	protein homolog model	phosphonic acid antibiotic	antibiotic inactivation	fosfomycin thiol transferase	fosfomycin
4	AAH450_16170	398	FosA8	55.88	protein homolog model	phosphonic acid antibiotic	antibiotic inactivation	fosfomycin thiol transferase	fosfomycin
3	AAH450_12305	1766	*Haemophilus influenzae* PBP3 conferring resistance to beta-lactam antibiotics	51.53	protein variant model	cephalosporin; cephamycin; penam	antibiotic target alteration	Penicillin-binding protein mutations conferring resistance to beta-lactam antibiotics	ceftriaxone; ampicillin; cefaclor; cefotaxime; cefditoren; cefdinir

Table S7. Cellular fatty acid composition (%) of ***E. psychrophila* sp. nov. P6884^T** and ***E. magellanica* sp. nov. P7711^T** strains and the closest phylogenetic relatives (*E. billingiae* CCM 9268^T, *E. endophytica* CCM 9270^T, *E. persicina* CCM 3799^T, *E. amylovora* CCM 1114^T, *E. pyrifoliae* NBIMCC 8386^T, *E. oleae* DSM 23398^T, *E. aphidicola* DSM 19347^T, and *E. phyllosphaerae* JCM 34792^T).

All data were taken from this study using cells grown to the late exponential phase (24h, except 48h for JCM 34792^T) on TSBA medium at 30 °C. TR, trace amount (< 1%); -, not detected

**Fatty acid**	**Strain Number**									
	**P6884^T**	**P7711^T**	CCM 9268^T	CCM 9270^T	CCM 3799^T	CCM 1114^T	NBIMCC 8386^T	DSM 23398^T	DSM 19347^T	JCM 34792^T
$C_{10:0}$ 3OH	-	-	-	-	-	-	-	-	-	3.9
$C_{12:0}$	2.9	5.4	2.8	4.2	4.0	5.1	4.7	8.1	4.1	4.1
$C_{12:0}$ 3OH	-	-	TR	-	-	-	-	-	-	3.3
$C_{13:0}$	TR	TR	1.0	TR	TR	TR	TR	TR	TR	-
$C_{14:0}$	4.8	2.8	4.7	4.7	5.3	4.8	5.2	TR	3.5	4.8
$C_{14:0}$ 2OH	-	-	-	-	-	-	TR	-	1.6	-
$C_{16:0}$	**25.1**	**27.2**	**24.5**	**30.1**	**31.0**	**33.5**	**35.1**	**36.4**	**32.3**	**33.5**
$C_{17:1}$ ω8c	TR	TR	1.2	TR	TR	TR	TR	TR	TR	-
$C_{17:0}$ cyclo	3.2	6.7	4.5	5.4	10.6	4.8	TR	4.1	7.7	4.8
$C_{17:0}$	1.5	2.6	6.8	2.7	2.2	2.9	TR	1.5	1.6	TR
Summed feature 1*	-	-	2.1	-	-	-	-	-	-	-
Summed feature 2*	10.2	9.9	8.9	9.7	10.0	10.0	9.1	10.1	10.1	9.0
Summed feature 3*	**35.6**	**27.9**	**31.0**	**26.5**	**25.6**	**28.4**	**36.9**	**29.2**	**27.0**	**21.1**
Summed feature 8*	15.1	15.3	10.7	14.5	7.9	8.1	6.6	12.7	9.5	12.8

* Summed features are groups of two fatty acids that cannot be separated by gas chromatography using the MIDI system.

Summed feature 1 contains $C_{15:0}$ iso H/$C_{13:0}$ 3OH; Summed feature 2 contains $C_{14:0}$ 3OH/$C_{16:1}$ iso I; Summed feature 3 contains $C_{16:1}$ *ω*7c/ $C_{16:1}$ *ω*6c and Summed feature 8 contains $C_{18:1}$ *ω*7c /$C_{18:1}$ ω6c.

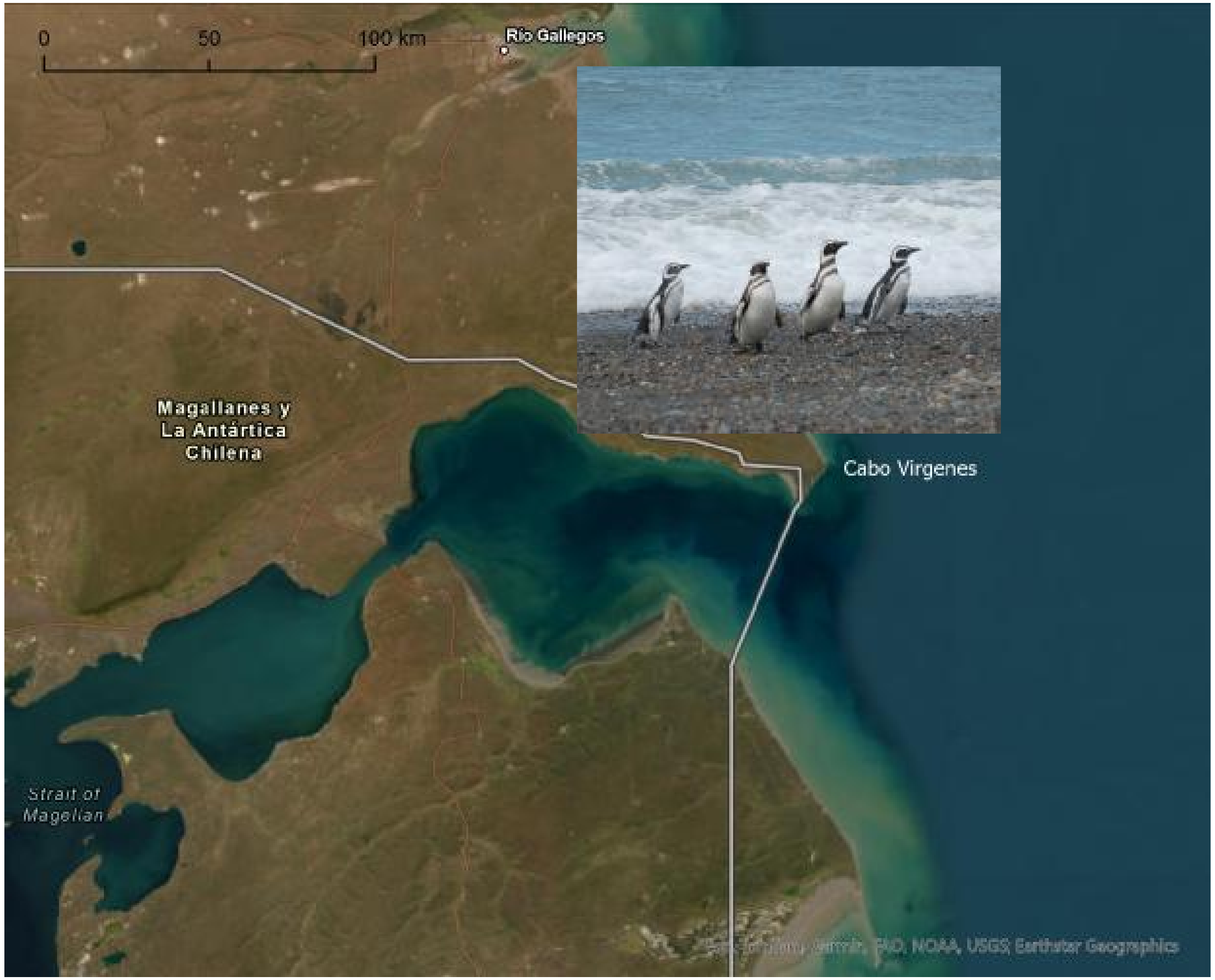

Figure S1. Magellanic penguins (*Spheniscus magellanicus*) on the seashore, Cabo Virgenes, Patagonia, Argentina.

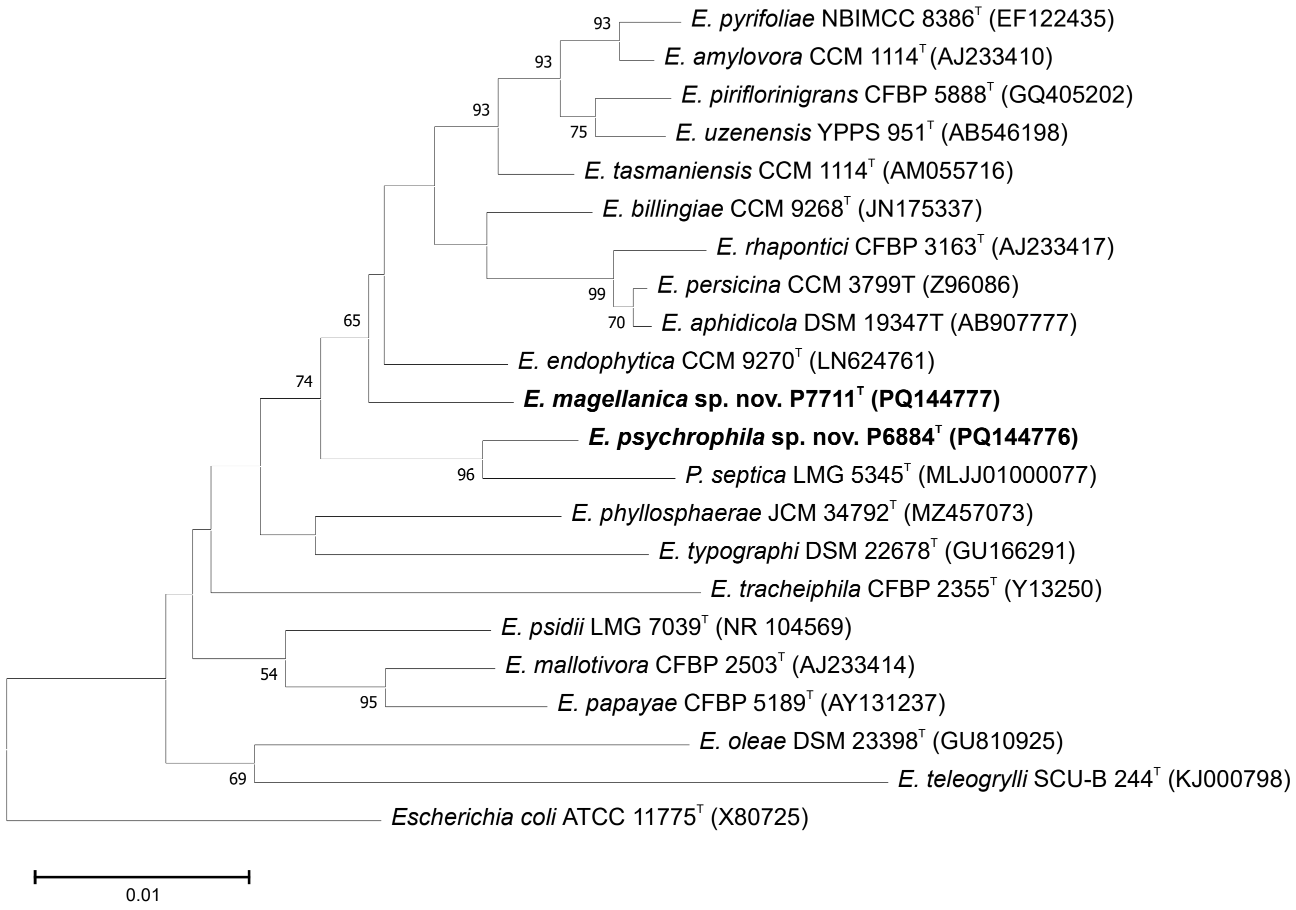

Figure S2.

Tree based on 16S rRNA gene sequence comparison showing the phylogenetic position of strains P6884^T and P7711^T and *Erwinia* and *Pantoae* reference strains. The evolutionary history was inferred using the Neighbor-Joining method [1]. The optimal tree is shown. The percentage of replicate trees in which the associated taxa clustered together in the bootstrap test (1000 replicates) are shown next to the branches [2]. The tree is drawn to scale, with branch lengths in the same units as those of the evolutionary distances used to infer the phylogenetic tree. The evolutionary distances were computed using the Kimura 2-parameter method [3] and are in the units of the number of base substitutions per site. All positions with less than 95% site coverage were eliminated, i.e., fewer than 5% alignment gaps, missing data, and ambiguous bases were allowed at any position (partial deletion option). There were a total of 1353 positions in the final dataset. *Escherichia coli* ATCC 11775^T was used as an outgroup. Bar 0,01 substitutions per nucleotic position.

1. Saitou N. and Nei M. (1987). The neighbor-joining method: A new method for reconstructing phylogenetic trees. Molecular Biology and Evolution 4:406-425.
2. Felsenstein J. (1985). Confidence limits on phylogenies: An approach using the bootstrap. Evolution 39:783-791.
3. Kimura M. (1980). A simple method for estimating evolutionary rate of base substitutions through comparative studies of nucleotide sequences. Journal of Molecular Evolution 16:111-120.

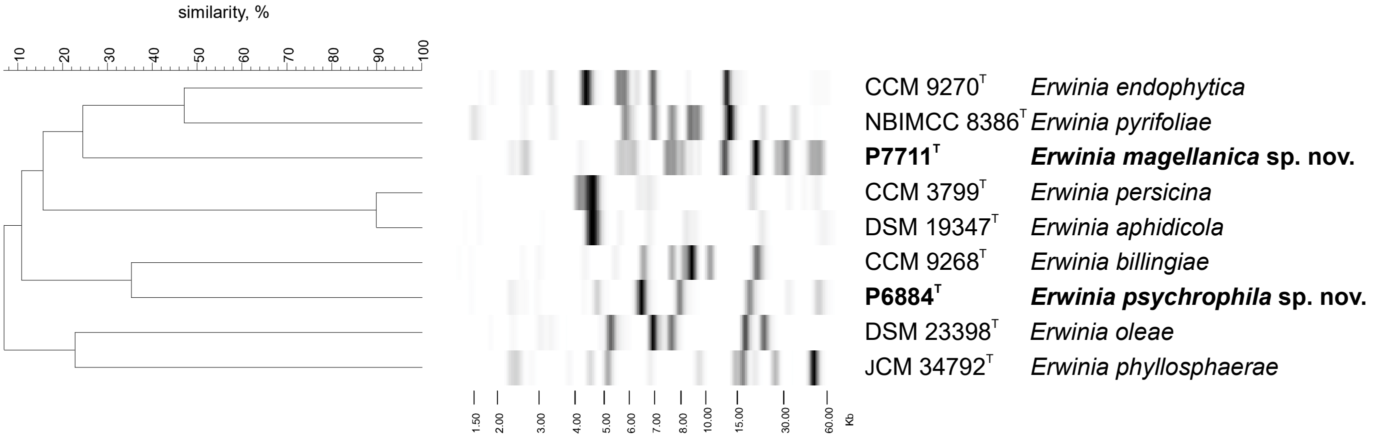

Figure S3. Dendrogram based on cluster analysis of *Eco*RI ribotype patterns obtained from *Erwinia* spp. strains using the RiboPrinter identification system. The dendrogram was calculated with Pearson's correlation coefficients with the UPGMA clustering method (r, expressed as percentage similarity values).
